# Cisplatin adducts of DNA as precursors for nanostructured catalyst materials†

**DOI:** 10.1039/d0na00528b

**Published:** 2020-08-24

**Authors:** Klaudia Englert, Ruba Hendi, Peter H. Robbs, Neil V. Rees, Alex P. G. Robinson, James H. R. Tucker

**Affiliations:** School of Chemistry, University of Birmingham Edgbaston Birmingham B15 2TT UK j.tucker@bham.ac.uk; School of Chemical Engineering, University of Birmingham Edgbaston Birmingham B15 2TT UK a.p.g.robinson@bham.ac.uk n.rees@bham.ac.uk

## Abstract

The synthesis and characterisation of novel metal-modified DNA precursors for fuel cell catalyst development are described. Material precursors in the form of metal–DNA complexes were prepared through the reaction of DNA with cisplatin at various loadings and spectroscopically tested to confirm the platinum binding mode and the degree of complexation. The surface morphology of the DNA–metal material was analysed by Scanning Transmission Electron Microscopy (STEM), which revealed the extent of platinum nanocluster formation, with low metal loadings leading to observation of individual platinum atoms. Electrochemical measurements showed a greater electrocatalytic activity for the hydrogen evolution reaction (HER) with increased platinum loadings, shifting the half wave potential, *E*_1/2_, away from the glassy carbon limit towards that of a bulk Pt electrode. This is explained further by Tafel plots, from which a change in the mechanism of the apparent rate limiting step for proton reduction from a Volmer to a Heyrovsky mechanism is postulated as the platinum loading increases.

## Abstract 2

A new role for the well-known cisplatin–DNA reaction is reported in the form of a method to generate a catalytic nanomaterial containing individually positioned platinum atoms.

## Graphical abstract

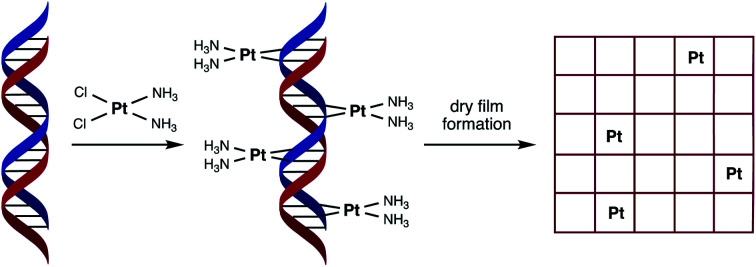

## Introduction

The electrolysis of water to form hydrogen *via* the Hydrogen Evolution Reaction (HER) and oxygen *via* the Oxygen Evolution Reaction (OER), is an environmentally friendly and sustainable way of generating chemical fuel.^1^ Through their recombination, chemical energy can then be converted into electricity, which is the fundamental operating principle behind Polymer Electrolyte Membrane Fuel Cell (PEMFC) technology, involving the hydrogen oxidation reaction (HOR) and oxygen reduction reaction (ORR).^2^ Platinum metal outperforms many electrocatalysts for both the HER (in acidic conditions) and ORR. However, given concerns over its cost and availability, there is ongoing research into how catalytic performance can be maintained with lower platinum loadings.^3–5^ Interestingly, one study found that lower loadings led to an improvement in efficiency.^6^ This was explained by an increase in platinum particle size (a function of increased loading) having an adverse effect on electrocatalysis. In fact metal nanostructure and size is an important consideration in catalyst design, as only certain metal sites on the surface of the catalyst are active, with the majority remaining inactive.^7^

**Fig. 1:**
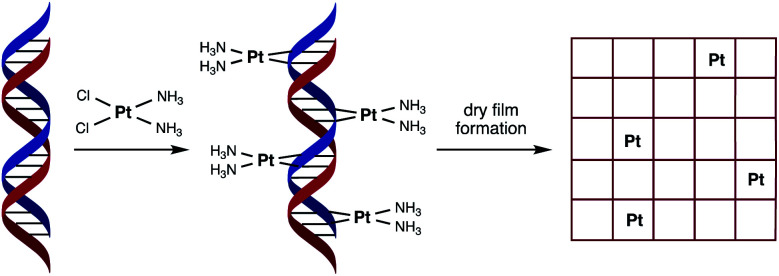
Schematic representation of the fabrication process of the catalytic material used in this study. Cisplatin binds covalently to duplex DNA, primarily at adjacent guanosine (G) nucleobases, *via* displacement of chloride by nitrogen heteroatoms. Platinum binding to each purine nucleobase (G or A) through a combination of mono- and bi-dentate adducts would result in a saturation stoichiometry of between 0.5 and 1 Pt atoms per base pair.

With the aim of reducing platinum metal loadings further in catalytic materials associated with clean fuel and energy production, we considered a novel synthetic biology approach to platinum nanostructure design involving the use of DNA as a scaffold for the accurate placement of metal atoms. In our design we hypothesised that spacing individual platinum atoms along the backbone of DNA would reduce undesirable clustering and expose more metal atoms as active sites, thereby raising the catalytic efficiency of the resultant material. The use of DNA as a green material and scaffold for catalytic metals is not unknown,^8,9^ having been previously used for attaching or directing the growth of platinum nanoclusters as catalysts for either the HER or ORR.^10–12^ However, our unprecedented approach to catalyst nanostructure design would involve using DNA to order the positioning of individual platinum atoms *via* the formation of multiple covalent adducts (Fig. 1).

In order to achieve this goal, we have turned to the anticancer drug cisplatin, [Pt(NH_3_)_2_Cl_2_], (cisPt) as a source of platinum atoms. Due to its medicinal importance, the binding mode of cisPt to duplex DNA has been extensively studied and is well understood, with the major interaction being the formation of two intrastrand Pt–N bonds that link adjacent guanine nucleobases at the N7 position, with other mono- and bi-dentate purine adducts also possible (Fig. 1).^13–16^ We considered its ability to form such strong covalent complexes with DNA as an important factor in ensuring the formation of robust catalyst precursor materials containing individually and regularly spaced platinum atoms. Herein we present our studies on complex formation between salmon milt DNA (sm-DNA)^17^ and cisPt in water and STEM microscopy and electrochemical studies on the resulting dry films formed from these metal–DNA complexes. Our studies reveal interesting trends in catalytic behaviour as a function of platinum loading in the HER reaction.

## Results and discussion

### DNA binding studies

In designing the metal–DNA precursor material, it was first important to establish the conditions for metal binding and the effect of different metal loadings. Hence a series of samples with different DNA : metal ratios were prepared by incubating mixtures of double stranded salmon milt DNA (sm-DNA), set at a fixed base-pair concentration of 1 mM, and various concentrations of cisPt overnight in Milli-Q water at 37 °C (Table 1).^18^ Buffers and salts were not used in the reaction mixture to prevent inhibition by chloride in the platination reaction and also to mitigate any adverse effect on the formation or study of the resulting dry films.

**Table 1:** Concentrations of cisPt used and its ratio per base pair (or nominal Pt loading, nPt) in relation to an unchanging concentration of sm-DNA in Milli-Q water (concentration = 1000 μM)

sm-DNA : cisPt ratio	Concentration of cisPt (μM)
1 : 0	0
1 : 0.1	100
1 : 0.25	250
1 : 0.5	500
1 : 1	1000
1 : 1.5	1500
1 : 2	2000

**Fig. 2:**
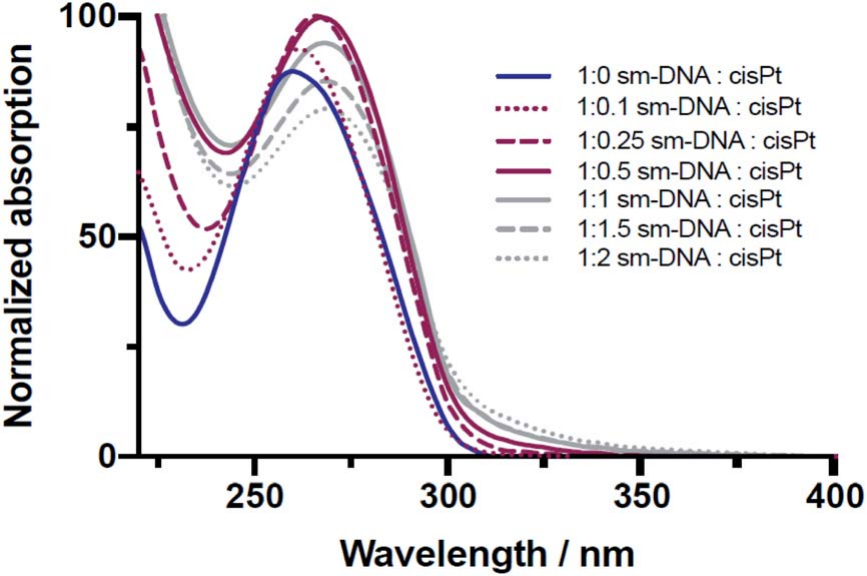
Overlaid UV/Vis spectra of sm-DNA samples with varying cisPt loadings (nPt) as equivalents per base pair in Milli-Q water.

The platination reaction with DNA was followed by UV/Vis spectroscopy and Circular Dichroism (CD) spectroscopy. As shown in the Fig. 2, the characteristic UV absorption band for DNA, centred at 260 nm, undergoes a gradual bathochromic shift of 260 nm to 269 nm, indicating the formation of multiple Pt-nucleobase covalent adducts, as previously reported.^19^ Interestingly an increase in the absorption intensity of this band was observed up to approximately 0.5 equivalents of cisPt per base pair (nPt = 0.5), followed by a decrease in intensity for samples with higher equivalents. Such a change from hyper- to hypochromicity indicates a change in binding mode, which can be rationalised if we consider that each DNA base-pair contains one purine nucleobase (*i.e.* either A or G) and therefore one potential metal binding site. These sites would accordingly be expected to become saturated in the presence of between 0.5–1 equivalents of cisPt per base pair, given that these interactions are comprised largely of mono- and bi-dentate Pt–N adducts (Fig. 1). The subsequent hypochromic effect at >1 equivalents, after all the purine heteroatom sites have been occupied, can be ascribed to unspecific binding interactions of a largely electrostatic nature between charged Pt(ii) complex ions and the negatively charged phosphodiester backbone of DNA.

**Fig. 3:**
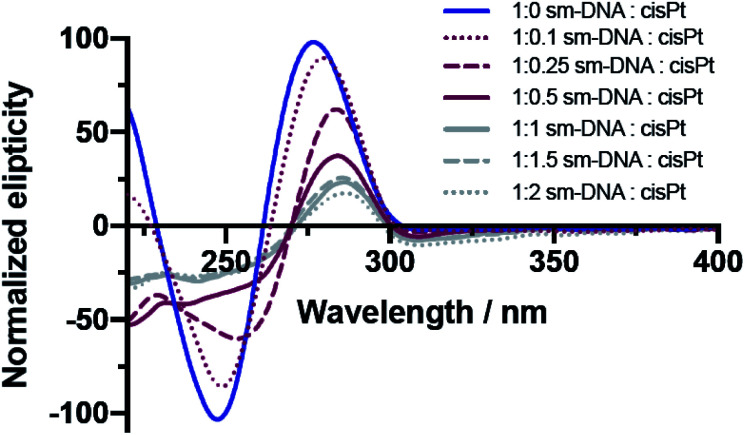
Overlaid CD spectra of sm-DNA samples with varying cisPt loadings (nPt) as equivalents per base pair in Milli-Q water.

The CD studies show significant changes to the characteristic B-DNA spectrum^20^ upon platination, with decreases in both the negative amplitude band (at 245 nm) and the positive amplitude band (at 280 nm) as shown in Fig. 3. As found with the UV/vis studies, two distinct trends are apparent; here the more dramatic changes to the CD spectrum are observed up until 0.5 equivalents of cisPt per base pair and to such an extent that the negative band has essentially disappeared at this loading level. Presumably the formation of extensive purine N–Pt adducts sufficiently kink the DNA so that the characteristic helicity within its tertiary structure is largely lost before the binding mode changes to interactions involving the phosphate backbone.^31–33^

### Microscopy

In order to study the morphology of the DNA–metal nanostructures and explore how the distribution of the platinum changes in the presence of DNA, a series of microscopy experiments were undertaken on films formed by drop casting from solution. Initially, AFM imaging was used to confirm the interaction between the DNA and cisPt under the incubation conditions employed. These studies indicated that the topography of the platinated DNA showed substantial differences to that of unmodified sm-DNA (see Fig. S1 in the ESI†). Scanning Transmission Electron Microscopy (STEM) imaging was then employed to achieve resolution at an atomic scale, which each sample prepared by casting 3 μL of the appropriate aqueous solution onto a holey carbon film on a 300 mesh copper TEM grid and drying under a lamp.

**Fig. 4:**
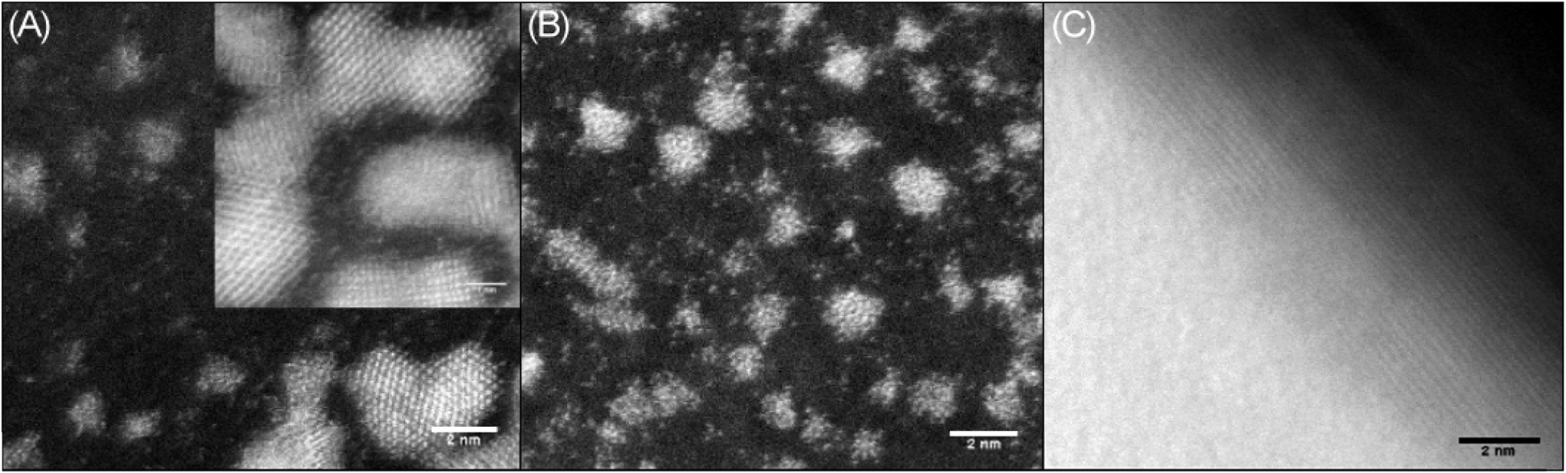
STEM images of drop cast films from 1000 μM solutions of (A) cisPt with magnification ×10 million (×20 million inset); (B) cisPt with magnification ×8 million; (C) sm-DNA with magnification ×8 million. All films cast from 3 μL solutions on holey carbon TEM grid.

Agglomerated nanoclusters of platinum were observed when cisPt was deposited directly from solution (*i.e.* without reaction with DNA first), as shown in Fig. 4A and B. The clusters were found to have a diameter of 1.81 ± 0.26 nm and 1.80 ± 0.27 nm for 1000 μM and 100 μM solutions of cisPt respectively (standard deviation from 27 and 29 measurements respectively, see Fig. S3 and Table S1 in the ESI† for more information). These findings are in good agreement with a previously reported^21^ value of 1.84 nm for metallic platinum clusters similarly formed from solutions of cisplatin. As expected the STEM image of pure DNA appeared as an amorphous carbon film (Fig. 4C).

**Fig. 5:**
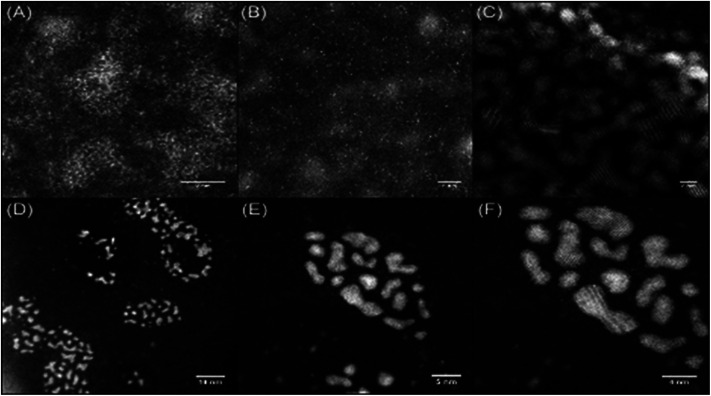
HAADF STEM images of drop cast films of (A) 1 : 0.1 sm-DNA : cisPt (equivalent to 100 μM cisPt) ×15 million; (B) 1 : 0.1 sm-DNA: cisPt (equivalent to 100 μM cisPt) ×8 million; (C) cisPt (from 100 μM solution, no DNA), ×6 million and (D–F) 1 : 1 sm-DNA : cisPt (equivalent to 1000 μM cisPt) at magnifications of 200k, 400k and 600k respectively. All films cast from 3 μL solutions on holey carbon TEM grid.

Keeping in mind the ultimate goal of producing hybrid materials consisting of localised and anchored single platinum atoms, HAADF STEM images were recorded for films formed from solutions of the DNA–metal complexes. Indeed it was found that the distribution of platinum was improved in the presence of DNA. This is depicted in Fig. 5A and B, where bright spots are observed for a base pair ratio of 1 : 0.1 (cisPt solution concentration: 100 μM, Table 1), indicating individual platinum atoms, as opposed to the agglomerated nanoclusters seen for cisPt deposited on its own at the same concentration (Fig. 5C). Interestingly, this dispersal effect is seen only for sub-stoichiometric values of added cisPt. As the platinum loading is increased in the Pt–DNA material, the effect is reversed and significantly larger nanoclusters are observed for a DNA : Pt base-pair ratio of 1 : 1 as shown in Fig. 5D–F (3.83 ± 1.85 nm, see Table S2 in the ESI†), compared to pure cisPt at the same concentration (1.81 ± 0.26 nm). Whilst it is not clear why the nanocluster size is larger in the case of highly loaded Pt–DNA complexes, it is apparent that cluster formation only predominates when metal loading exceeds the available nucleobase binding sites on the DNA, *i.e.* at the point that non-specific cisplatin interactions can occur with the DNA backbone, as indicated by the spectroscopic studies (Fig. 2 and 3).

### Electrochemical behaviour

The electrochemical behaviour of the various metal–DNA samples was then investigated using the hydrogen evolution reaction (HER) in an acidic environment (pH = 3). Despite the largely resistive nature of polymeric DNA,^22^ additional redox-active centres retain their electrochemical activity^23,24^ and DNA saturated with transition metal cations can be electrochemically reduced to metallic nanowires.^9,25,26^ Solutions of the DNA–cisPt adducts (25 μL) were drop cast onto the GC surface (*d* = 3 mm) to form the working electrode (WE) and a series of cyclic voltammetry tests were then run at 50 mV s^−1^ in 1 mM HClO_4_, 100 mM NaClO_4_.

**Fig. 6:**
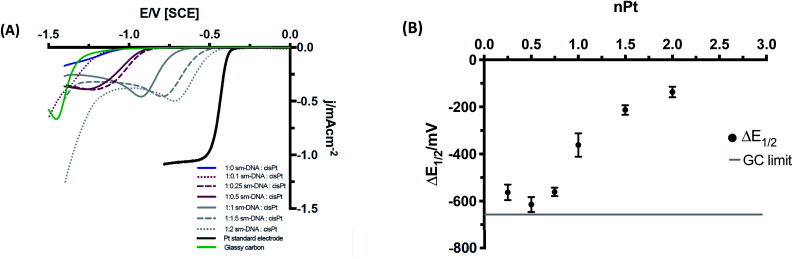
(A) Cyclic voltammograms of Pt–DNA modified GC at 50 mV s^−1^ in HClO_4_ and NaClO_4_ (1 and 100 mM respectively, 10^th^ cycle shown in each case). Stable and consistent voltammograms were observed from the 3^rd^ cycle and beyond. The Pt standard electrode included for reference was a microelectrode (see main text). (B) Changes in half wave potential (Δ*E*_1/2_), with nominal loading of platinum metal, nPt (equivalents per base pair), where Δ*E*_1/2_ = *E*_1/2_ (Pt–DNA sample) − *E*_1/2_ (Pt electrode).

The DNA-modified electrodes show a clear correlation between the nominal Pt loading (nPt) and voltammetric response as shown in Fig. 6A in comparison to a bulk Pt microelectrode (whose current does not scale with electrode area, hence it has a higher current density than the Pt–DNA modified macroelectrodes). The half wave potential (*E*_1/2_) can be employed as a first approximation method to provide an indication to the formal potential of the material (please see ESI† for more details). Fig. 6B shows the variation in the half potential shift with Pt loading, where Δ*E*_1/2_ = *E*_1/2_(sample) − *E*_1/2_(bulk Pt). As the nominal platinum loading is increased, Δ*E*_1/2_ first shifts to larger values (towards the GC limit) until approximately nPt = 0.5, then moves away from the GC limit and towards the bulk platinum behaviour. We note that a value of nPt = 0.5 corresponds to the limit of specific binding of cisPt to the DNA: above this ratio, additional Pt is expected to be non-specifically bound to DNA, and hence less tightly bound to the structure. We postulate that this is not coincidental, with individually bound Pt atoms present below nPt = 0.5, (although possibly not exclusively single atoms) whereas above this value, the formation of small clusters and nanoparticles that are nucleated at the bound Pt atoms will become more prevalent.

The shift in the position of the reduction wave, and changes in its shape as loading increases, can be due to different factors. First, the highly resistive nature of the DNA-modified electrode will cause a considerable shift to more negative potentials. As Pt is added to the DNA, these atoms are not all in electrical contact with the substrate electrode and their contact resistances are not the same. As Pt loading is increased, the mean contact resistance might be expected to decrease, causing an ohmic shift in the wave towards lower overpotentials. Secondly, as the Pt loading increases, the spatial distance between Pt atoms/clusters decreases and the diffusional characteristics may change from category 1 at ultra-low loadings, *via* categories 2 & 3 to category 4 at moderate loadings as defined by Davies *et al.*^27^ This leads to a gradual change of shape of the voltammetry from sigmoidal to peak-shaped. At the loadings used here, and based on the STEM imaging above, the diffusional behaviour is expected to be within category 4. Similarly, this change in shape may also reflect the mechanistic changes occurring as Pt loading increases (see below).

### Mechanistic analysis

To further compare the electrocatalytic behaviour at the different DNA–cisPt loadings explored, a Tafel analysis was employed to reveal the reaction mechanism of the hydrogen evolution reaction (HER). Here it should be noted that a Tafel treatment of these modified electrodes is necessarily approximate due to the likelihood that the voltammetry is influenced by kinetic, mass transport and ohmic (resistance) factors. The mechanism for the HER in acidic conditions has been well studied and reported in the literature over the years^2,28–30^ and has been outlined to involve two of three possible steps. The first step involves the reduction of a proton on an active site of the catalyst surface, the Volmer step eqn (1). Molecular H_2_ is then evolved either by a second proton/electron transfer, the Heyrovsky step, eqn (2), or through the recombination of two adsorbed protons at the surface, the Tafel step, eqn (3).

In these equations, * denotes a vacant active site on the surface of the catalyst and H* is a hydrogen atom adsorbed on an active site.

1 Volmer: H_3_O^+^ + e^−^ + * ⇌ H* + H_2_O

2 Heyrovsky: H* + H_3_O^+^ + e^−^ ⇌ H_2_ + H_2_O

3 Tafel: 2H* ⇌ H_2_

**Fig. 7:**
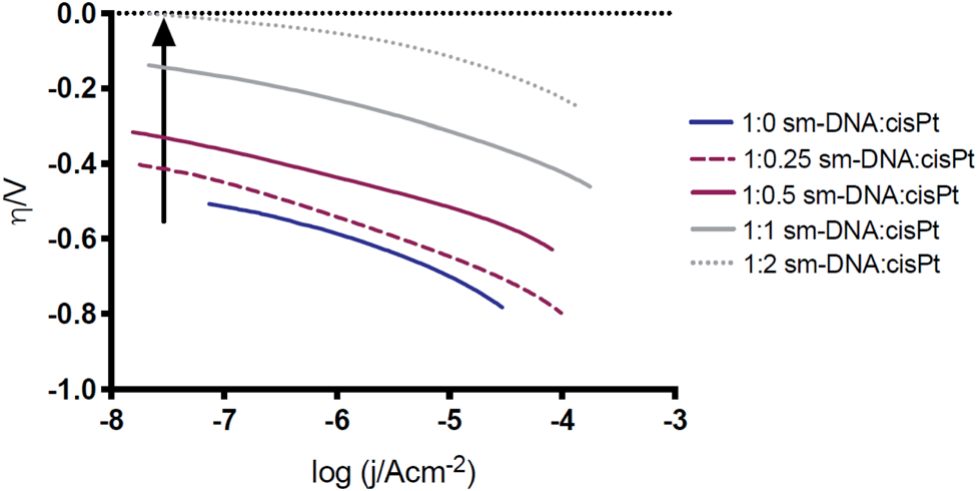
Comparison of the Tafel plots for a selection of processed Pt–DNA films. The arrow denotes increasing Pt loading.

Tafel plots (Fig. 7) were constructed from the electrochemical data obtained in Fig. 6A and the Tafel slope was consequently found from the gradient of the plot, for the different DNA : cisPt loadings employed. The Tafel slopes found were compared to the widely accepted and reported theoretical limits as the rate determining reactions, for the Volmer, Heyrovsky and Tafel steps (120, 40 and 30 mV dec^−1^ respectively). These are found at both a high and low over potential (*η*), see Fig. 7. Commonly platinum disks and high surface area catalysts have shown a Tafel limiting step (slope of −30 mV dec^−1^). Detailed analysis of this is discussed in the literature.^2,28,29^ As the Pt loading increases, the magnitude of the Tafel slope tends towards that of bulk platinum (Tafel slope of −40 mV dec^−1^) and at a high *η* the behaviour tends towards the GC limit (Tafel slope of −120 mV dec^−1^). This is in line with the observations made with the trend of the half wave potential (*E*_1/2_) with cisPt loading.

**Fig. 8:**
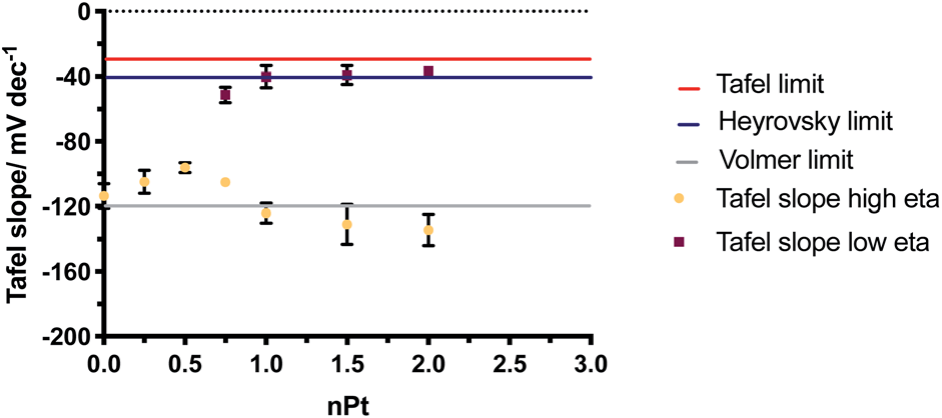
Change in Tafel slopes for increasing nPt at low (squares) and high (circles) overpotential (*η*). The theoretical Volmer, Heyrovsky and Tafel limits are displayed.

The changes in the Tafel slope with the loading of platinum shown in Fig. 8, reflect the change in the reaction mechanism *i.e.* from the Volmer limit to the Heyrovsky. This implies that as the Pt loading is increased, the rate-limiting step for proton reduction changes from that described in eqn (1) to eqn (2). The Tafel slope remains the same at high and low *η* for low nominal platinum loadings, *i.e.* at nPt < 0.5. Whereas at nPt > 0.5, the more familiar behaviour seen in bulk Pt is observed, with different Tafel slopes for high and low overpotentials, providing further evidence that cluster formation of Pt is increasingly found beyond nPt = 0.5.

From an electrochemical standpoint it has previously been estimated that a minimum of five atoms of platinum are required to electrolyse acidic solutions.^31^ This suggests that at lower Pt loadings of *ca.* 0.5 and below, where nucleobase coordination dominates, the DNA: cisPt films may not reduce to form large enough clusters, due to the increased interparticle distance between the fewer platinum catalyst sites. At these low loadings, the Pt atoms and few-atom clusters experience a high resistance, and the potential on these nanoclusters will be less negative than the potential on the underlying electrode. The resistivity of the DNA connection to the substrate electrode is therefore likely to be a significant controlling factor in the HER kinetics. However, at higher nominal loadings, where the cisPt species coordinates to the DNA non-specifically^32–34^ the metal sites would be both more numerous and more mobile than covalently bound moieties, bringing them in to close enough proximity to form catalytic centres in irregular arrays of metallic particles. As the size and number of the Pt clusters increase, the electrical contact to the substrate electrode will lower, as will the potential (ohmic) drop across it.

## Conclusions

In conclusion, we have demonstrated a new role for the well-known cisplatin–DNA reaction in generating catalytic nanomaterials, in which the DNA acts as a scaffold to disperse platinum atoms along its backbone. Through STEM imaging, it was found that the surface morphology of drop cast Pt–DNA films changed as a function of the platinum content, with ultra-low loadings reducing clustering effects to the extent that individual platinum atoms could be observed. In contrast, at higher metal loadings, that is at a DNA : Pt base-pair ratio of 1 : 1 and above, the effect was reversed and larger nanoclusters were observed in the presence of DNA compared to in its absence. This implies that there is an optimum ratio of platinum metal loading to DNA for studying electrocatalysis. This new approach of using DNA as a scaffold to fine-tune the loading and distribution of individual metal atoms shows promise for the development on new electrocatalytic materials, as reflected by the electrochemical data and the analysis of the reaction mechanisms through the Tafel plot constructs. The observations of different behaviour in the ranges of nPt < 0.5 and nPt > 0.5 suggest that further insight is to be gained from studying this system at ultra-low Pt loadings. Future work will accordingly focus on this aspect in the interest of designing lighter and cheaper materials for fuel cell electrocatalysis.

## Experimental section

### Materials and reagents

Sodium perchlorate (≥98%), stock of sm-DNA in a form of sodium salt, and cisPt (99.99% purity) were all purchased from Sigma Aldrich. Perchloric acid (Trace Select Ultra, 67–72%) was purchased from Fluka Analytical. All electrolytes were made up with deionised (DI) water of resistivity no less than 18.2 MΩcm (Milli-Q, Millipore) and thoroughly degassed with dry nitrogen (oxygen-free, BOC Gases plc) prior to experimentation.

### Synthesis and characterisation of Pt DNA adducts

sm-DNA was dissolved in Milli-Q water and sonicated to make a stock solution. The concentration of the stock solution was determined by UV/Vis spectroscopy with the epsilon = 6600 M^−1^ cm^−1^ taken from the literature.^1^ The desired concentrations of DNA and cisPt solutions (Table 1) were combined together by pipetting. The samples were heated overnight for at least 10 h at 37 °C and monitored by UV/Vis spectroscopy. The samples were analysed using Shimadzu UV-1800 spectrophotometer, Jasco J-810 spectropolarimeter and Cary 5000 UV-Vis-NIR Spectrophotometer.

### Electrochemical measurements

A three-electrode cell was employed, using a glassy carbon electrode (GC, *d* = 3 mm, BASi) as the working electrode, a saturated calomel (SCE, BASi) reference and a bright platinum mesh counter electrode. The Pt disk electrode was a Pt microelectrode (*d* = 10 μm, ALS). The cell was controlled by an Autolab 128N potentiostat running Nova 2.1 software (Metrohm-Autolab BV, Netherlands). All potentials are reported against a SCE, all supporting electrolytes were 0.1 M. The glassy carbon (GC) electrodes were polished on microcloth pads using an alumina slurry in a decreasing order of size (1.0, 0.3, 0.05 μm, Buehler IL), followed by cleaning for 60 s in an ultrasonic bath and drying under a gentle flow of nitrogen. Once dry, the electrodes were modified by casting an aliquot of DNA : cisPt (25 μL cm^−2^) and drying under a lamp (30 cm elevation). The Pt–DNA films were cycled at 50 mV s^−1^ for 10 cycles, between 0 and −1.4 V, until a stable proton reduction wave was observed. The 10^th^ cycle was used for analysis and discussion.

### STEM-HAADF

The surface morphology of the material was attained using Scanning Transmission Electron Microscopy (STEM) operated in both Dark field and Bright field imaging using a JEOL2100F instrument. The STEM was operated in Z-contrast mode using a HAADF detector at 200 keV acceleration voltage. All stage alignment was achieved using gold nanoparticles on a Cu–carbon TEM grid, as detailed in the ESI (Fig. S2†).

## Conflicts of interest

There are no conflicts to declare.

## Supplementary Material

NA-002-D0NA00528B-s001
