# Supplementary material for: Cisplatin adducts of DNA as precursors for nanostructured catalyst materials
Source: Nanoscale Adv. 2020 Aug 24;2(10):4491–7. doi: 10.1039/d0na00528b (PMC9417135; doi:10.1039/d0na00528b)
Supplement: NA-002-D0NA00528B-s001 [file NA-002-D0NA00528B-s001.pdf]

Electronic Supplementary Material (ESI) for Nanoscale Advances.
This journal is © The Royal Society of Chemistry 2020

Supplementary Information

Cisplatin adducts of DNA as precursors for nanostructured catalyst materials

Klaudia Englert,[a] Ruba Hendi,[b] ,Peter H. Robbs,[b] Neil V. Rees,[b*] Alex P. G. Robinson[b*] and James H. R. Tucker,[a*]

[a.]School of Chemistry, University of Birmingham, Edgbaston, Birmingham B15 2TT, UK.
[b.]School of Chemical Engineering, University of Birmingham, Edgbaston, Birmingham B15 2TT, UK

Surface Topography

The change in the surface topography as a result of the interactions of the cisplatin with DNA were observed using 'Dimension 3100' Atomic Force Microscope (AFM) (Veeco Digital Instruments, Bruker), equipped with a NanoScope IIIa controller. 20 µl of the sample was drop casted onto a Highly Ordered Pyrolytic Graphite (HOPG) surface and was left to dry in air before using a stream of Nitrogen gas. The microscope was operated in tapping mode.

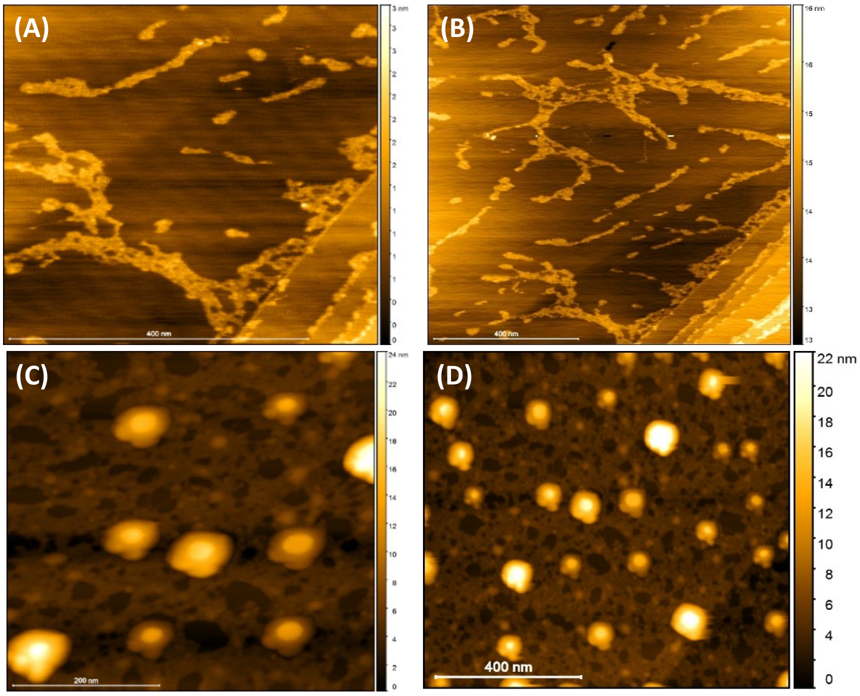

Fig.S1 - AFM Imaging (A, B) 1000 µM sm-DNA (C, D) 1:1 sm-DNA:cisPt on Highly Ordered Pyrolytic Graphite (HOPG)

There is an observable change in the surface topography of DNA upon its interaction with cisplatin. This entails the transition of the extended DNA chains to more compact particles, Fig.S1 A&B. This observation has previously been reported in the literature[1], and the observable changes in the DNA topography are attributed to the bending of the DNA chain upon complexation with cisPt. The change is an indication of the co-ordination of the drug with the purine bases of the DNA[1]. Experimental factors such as incubation time of the cisplatin with the DNA can have an influence on the extent of bending towards the major groove[1].

STEM Stage Alignment

Initially, gold nanoparticles deposited on a carbon TEM grid are used to achieve the alignment of the stage on the JEOL2100F instrument. Once the ronchigram is attained, images of high resolution can be recorded, see fig.S2 below.

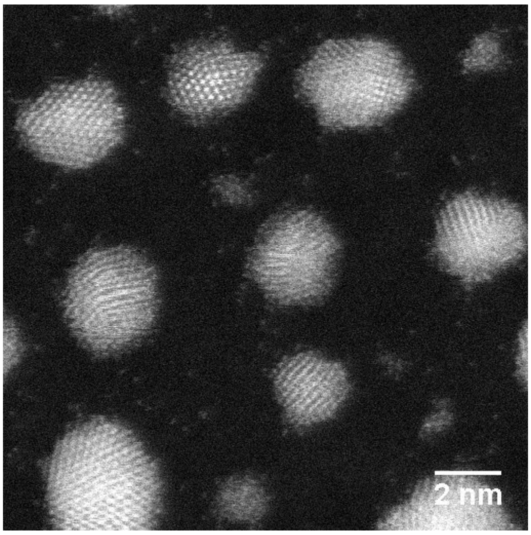

Fig. S2- HAADF STEM imaging of standard alignment sample of the microscope made of sputtered gold (Au) nanoparticles on a carbon TEM grid (x 10 million)

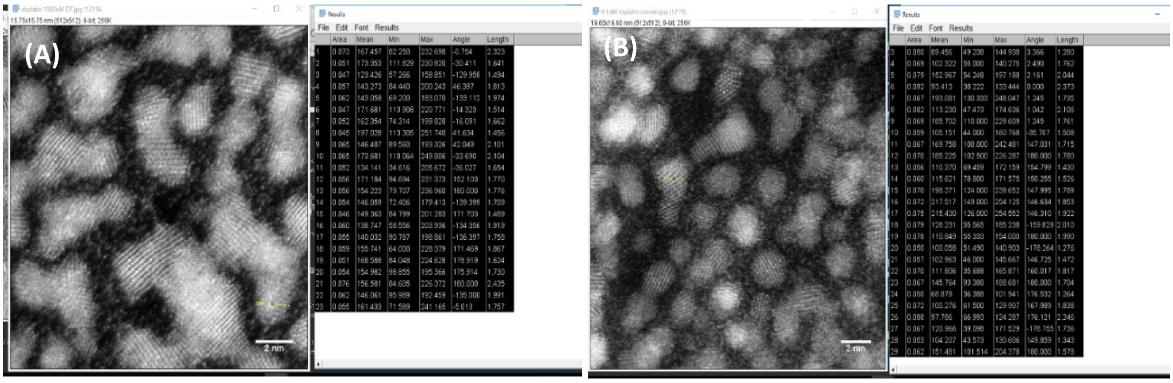

Fig. S3 - Diameter measurements made using tools in 'Image J' imaging software of platinum nanoclusters observed in drop cast films of (A) 1000 µM cisPt solution (B) 100 µM cisPt solution

Table S1- Diameter size measurements using tools in "Image J' for drop cast films of cisPt (concentrations in water of 100 and 1000 µM)

**Measurement number**	**100 µM cisPt**	**1000 µM cisPt**
	**Diameter size/ nm**	
1	2.093	2.323
2	1.863	1.641
4	1.762	1.494
5	2.044	1.813
6	2.373	1.974
7	1.735	1.514
8	2.106	1.662
9	1.761	1.456
10	1.508	2.101
11	1.715	2.104
12	1.78	1.654
14	1.526	1.77
15	1.789	1.776
16	1.853	1.709
17	1.922	1.489
18	2.01	1.919
19	1.99	1.758
20	1.276	1.867
21	1.472	1.624
22	1.817	1.73
23	1.704	2.435
24	1.264	1.991
25	1.838	1.757
26	2.246	-
27	1.736	-
29	1.578	-
**Average (3 significant figures)**	1.80	1.81
**STDEV ERROR (3 significant figures)**	0.27	0.26

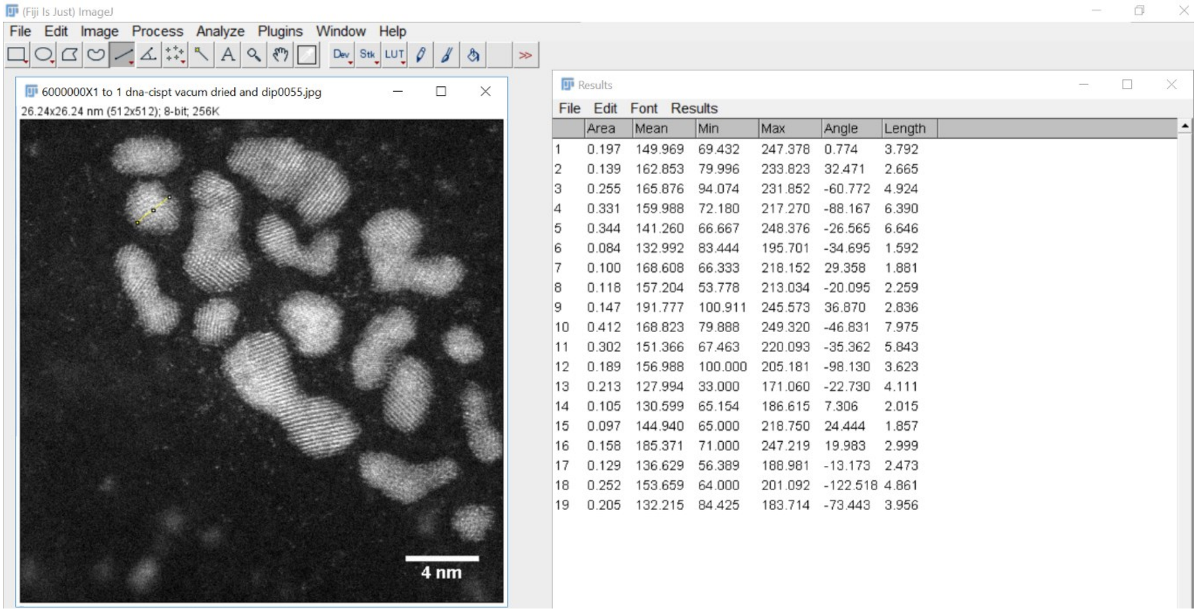

Fig. S4- Nanocluster diameter size measurements in 'Image J' of the 1 : 1 sm-DNA : cisPt solution drop cast on holey carbon TEM grid

Table S2- Diameter size measurements using tools in 'Image J' for drop cast films of 1 : 1 sm-DNA : cisPt displayed in Fig. S4

**Measurement number**	**Diameter size/ nm**
1	3.79
2	2.67
4	4.92
5	6.39
6	6.65
7	1.59
8	1.89
9	2.26
10	2.83
11	7.98
12	5.84
14	3.62
15	4.11
16	2.01
17	1.86
18	3.00
19	2.47
**Average (3 significant figures)**	3.83
**STDEV ERROR (3 significant figures)**	1.85

Electrochemistry

1. Use of half-wave potentials

For a general one-electron transfer reaction A + e- —— B

the half wave potential of a reversible steady-state voltammogram (i.e. measured at a microelectrode) is given by [2]:

$$E_{1/2,rev} = E_f^0 + \frac{RT}{F} ln \frac{D_B}{D_A}$$

and for an irreversible steady-state voltammogram it is:

$$E_{1/2,irrev} = E_f^0 + \frac{RT}{\alpha F} ln \frac{k_0 r}{D_A}$$

Where E_f^0 is the formal reduction potential, D_A and D_B are the diffusion coefficients of A and B respectively, α is the transfer coefficient, k_0 is the standard heterogeneous electron transfer rate constant, and F, R and T have their usual meanings.

In contrast, at macroelectrodes the half-wave potentials (actually the half-peak current potentials, $E_{p/2}$) for reversible and irreversible processes are given by [3]

$$E_{p/2,rev} = E_f^0 + 1.09 \frac{RT}{F}$$

$$E_{p/2,irrev} = E_f^0 + \frac{RT}{\alpha F} ln \left(\frac{2.936 k_0}{\sqrt{\alpha}} \right)$$

References

1. Z. Liu, S. Tan, Y. Zu, Y. Fu, R. Meng, Z. Xing, *Micron.* 2010, **41**, 833–839.

2. A.M.Bond, K.B. Oldham, C.G. Zoski, *Anal. Chim. Acta* 1989, 216, 177-230

3. J. Gonzalez, A. Molina, F. Martinez-Ortiz, M. Lopez-Tenes, R.G. Compton, Electrochmi. Acta 2016, 213, 911-926
